# *Hyrtios* sp.-associated *Cladosporium* sp. UR3 as a potential source of antiproliferative metabolites

**DOI:** 10.1186/s12866-024-03560-6

**Published:** 2024-11-01

**Authors:** Omnia Hesham Abdelhafez, Abeer H. Elmaidomy, Mohamed Hisham, Stefanie P. Glaeser, Peter Kämpfer, Jun Wu, Usama Ramadan Abdelmohsen

**Affiliations:** 1 Department of Pharmacognosy, Faculty of Pharmacy, Deraya University, New Minia City, Minia Egypt; 2 Department of Pharmacognosy, Faculty of Pharmacy, Beni-Suef University, Beni-Suef, 62514 Egypt; 3 Department of Pharmaceutical Chemistry, Faculty of Pharmacy, Deraya University, New-Minia, 61512 Egypt; 4 Institute of Applied Microbiology, Justus-Liebig University Gießen, Gießen, Germany; 5 Guangdong Key Laboratory for Research and Development of Natural Drugs, College of Pharmacy, Guangdong Medical University, Dongguan, 523808 China; 6 Department of Pharmacognosy, Faculty of Pharmacy, Minia University, Minia, 61519 Egypt

**Keywords:** Sponge, Fungi, *Hyrtios*, Cytotoxicity, Metabolomics

## Abstract

**Background:**

Sponge-associated microorganisms are promising resources for the production of bioactive compounds with cytotoxic potential. The main goal of our study is to isolate the fungal endophytes from the Red Sea sponge *Hyrtios* sp. followed by investigating their cytotoxicity against number of cell lines.

**Results:**

The fungal strain UR3 was isolated from the Red Sea sponge using Sabouraud dextrose agar media. It was identified based on partial 18 S rRNA gene and ITS sequence analyses as *Cladosporium* sp. UR3. The in vitro cytotoxic potential of the ethyl acetate extract of the fungal isolate was evaluated using MTT assay against three cancer cell lines: CACO2, MCF7, and HEPG2. Metabolomics profiling of the obtained ethyl acetate extract using LC-HR-ESI-MS, along with molecular docking and pharmacological network studies for the dereplicated compounds were performed to explore its chemical profile and the possible cytotoxic mechanism of the sponge-associated fungi.

**Conclusion:**

These results highlighted the role of sponge-associated fungi as a fruitful resource for the discovery of cytotoxic metabolites.

**Supplementary Information:**

The online version contains supplementary material available at 10.1186/s12866-024-03560-6.

## Introduction

Cancer remains one of the most lethal disease worldwide [1]. It is caused by uncontrolled cell division, abnormal cell growth as well as gene mutations [2]. Numerous factors such as lifestyle, nutrition, continuous exposure to radiation, and smoking are the main causes of carcinogenesis [3]. It is reported that the current rate for developing cancer is 1 to 5 in men and 1 to 8 in women. Additionally, the most common cancer types include lung cancer, breast cancer, prostate cancer, colorectal cancer, stomach cancer, and liver cancer [4]. There are major side effects of the currently available chemotherapeutic regimens as they affect the rapidly dividing tissues and cells such as hair follicles, gastrointestinal tract cells, and bone marrow. Additionally, these medications also responsible for the development of cancer stem cells and multi-drug-resistance [5]. Therefore, there is a great demand for the discovery of natural-derived chemotherapeutic agents with less side effects.

Natural products with their large chemical diversity have proven to be a fertile field of potential therapeutic candidates [6]. There is always a continuous demand for the discovery of novel natural bioactive metabolites due to the rise of cancer and infectious outbreaks [7–10]. Different classes of metabolites, such as alkaloids, flavonoids, and terpenoids are all obtained from different natural resources [11]. These metabolites exhibited various bioactivities as anti-inflammatory, anti-oxidant, neuroprotective, and anticancer potential [11]. Since the past decades, great efforts have been made to isolate natural metabolites with anticancer potential. These tremendous efforts had led to the discovery of a great number of anticancer drugs [12]. Many natural compounds can interfere directly with the main signaling pathways incorporated in the carcinogenesis [13]. Till now, some of those anticancer drugs still the mainstay approaches in cancer therapy, such as vincristine, paclitaxel, etoposide, which were obtained from plants, while, actinomycin D, mitomycin C, and bleomycin, were obtained from bacteria and marine resources, respectively [14]. On the other hand, marine-sponge is considered as a promising source of bioactive metabolites with unique scaffolds [15–18]. They showed different bioactivities as antibacterial, antiviral, anticancer, anti-inflammatory, and immunosuppressive agents [12].

Oceans biodiversity represent nearly 50% of all the globes biodiversity [19]. Accordingly, marine microorganisms are considered as a sustainable resource of diverse bioactive molecules [20]. They are also a promising source of kinase and proteasome inhibitors which are in clinical trials for cancer therapy [21]. Marine-associated fungi produce a wealth of secondary metabolites, which are bioactive with pharmaceutical importance [22]. Besides, some marine fungal-derived metabolites met the criteria for formulating suitable pharmaceuticals as they showed suitable oral bioavailability, and appropriate physico-chemical properties [23]. The genus *Cladosporium* is renowned as a fruitful resource of different classes of metabolites and for its implication in medical aspects [24]. They produce different metabolites such as alkaloids, pyrones, macrolides, fatty acids, terpenes, sterols, lactones, and tetramic acid derivatives [25]. They also showed various bioactivities as antimicrobial, antioxidant, cytotoxic, insecticidal [26].

In view of this point, our current research aims to describe the isolation and identification of a fungal strain associated with the Red Sea sponge *Hyrtios* sp. The cytotoxic potential of the fungal strain was investigated against three cancer cell lines. Moreover, the ethyl acetate extract of the fungi was explored by LC-HRMS-based metabolomics. The identified compounds were afterward subjected to in silico molecular docking and pharmacological network analyses.

## Materials and methods

### Sponge material

The sponge biomass used during our work was previously collected by Safwat Ahmed (Suez Canal University) from Sharm el-Sheikh, Red Sea, Egyptian coasts by scuba diving at a depth of 9.14 m. The collected biomass was frozen and stored at − 20 °C till investigation. The biomass was identified by van Soest (Institute of Systematic Population Biology, Amesradam University, The Netherlands). A voucher specimen (Dr-Ph-01) was deposited at the Department of Pharmacognosy, Faculty of Pharmacy, Deraya University, Egypt.

### Isolation and purification of fungal strains

After collecting the sponge biomass, it was twice cleaned with sterile saltwater, dried, and then bathed in 70% ethanol for a minute or two to surface sterilise it before being allowed to air dry. Furthermore, the inside tissues of the sponge were divided into tiny sections, each measuring 0.5 cm^3^, using a sterile knife in a sterile environment. The tiny segments were surface streaked on Sabouraud dextrose agar plates (the SDA were dissolved in sea water and given with amoxicillin and flucloxacillin 0.05 g/L to suppress bacterial growth). For a maximum of two weeks, the plates were incubated at 28 °C, and any growth was regularly observed. The fungi were taken out of their hyphal tips and placed on a new Sabouraud dextrose agar medium. Duplicate plates were manufactured to lower the risk of contamination. Subcultures were carried out repeatedly until pure isolates were gathered. For every isolate, morphological identification was performed [27, 28].

### Molecular identification and phylogenetic analysis of fungal strain UR3

For molecular identification and phylogenetic analysis, genomic DNA from fresh fungal biomass scraped from agar plates was extracted using the MasterPure Yeast DNA extraction kit (epicenter, Madison, Wisconsin). The internal transcribed spacer (ITS) region, which contains ITS 1, the 5.8 S ribosomal RNA gene, and ITS 2, was amplified and sequenced using the Sanger sequencing technology, as well as the small subunit ribosomal RNA gene (18 S rRNA gene). ITS-4 (5´- TTCCTCCGCTTATTGATATGC-3´) and NS1 (5´-GTAGTCATATGCTTGTCTC-3´) were the primer systems employed for this amplification [29]) Using MEGA11 version 11.0.13, the sequences were manually corrected [30]. The ITS region and the small subunit ribosomal RNA gene were individually uploaded to GenBank and assigned Accession Numbers OR900633.1 and OR900731.1. The fungal type material with the highest sequence similarities was found using the National Centre for Biotechnology Information (NCBI) Nucleotide BLAST and the rRNA/ITS NCBI RefSeq curated databases for the appropriate DNA sections (Bioproject PRJNA224725; updated 2023-12-19). The ITS and 18 S rRNA gene sequences (type material) of the subsequent related fungal strains were obtained from the NCBI and added to MEGA 11. The sequences were aligned using ClustalW. After manually adjusting the alignments, the Kimura 2-parameter model was used to generate Maximum Likelihood trees [31]. The first tree or trees for the heuristic search were automatically produced by using the Neighbor-Join and BioNJ algorithms on a matrix of pairwise distances computed using the Maximum Composite Likelihood (MCL) technique. The next topology selected was the one with the highest log probability value. For the 18 S rRNA gene sequence analysis, the final dataset has 59 sequences and 1,143 nucleotide positions; for the ITS sequence analysis, it contains 71 sequences and 666 nucleotide positions.

### Cultivation of pure fungal strain UR3 and extraction of fungal culture

For the purpose of conducting cytotoxic activity testing and LC/MS chemical profiling, the pure, isolated fungal strain UR3 has been produced. The isolated fungal strain *Cladosporium* sp. UR3 was fermented using the solid-state fermentation protocol [10, 32, 33]. In this method, 150 µl of the isolated fungal strain was inoculated and streaked over ten solid plates containing the media Sabouraud Dextrose Agar (10 g peptone, 40 g dextrose, and 20 g agar prepare in 1 L. distilled water). The plates were kept at 30 °C for 10 days for their optimum growth. After that, the plates were chopped into small pieces and macerated in flasks containing ethyl acetate (300 ml each) to obtain most of secondary metabolites produced during the fermentation process. Finally, the extract was prepared by evaporating the ethyl acetate solvent using rotary evaporator (Heidolph^®^, 45 °C, 154 r.p.m).

### Cytotoxic potential

The cytotoxic potential of the ethyl acetate extract of *Cladosporium s*p. UR3 was evaluated using the MTT assay against three human tumour cell lines: HEPG2 hepatocellular carcinoma (ATCC NO. HB-8065), CACO2 colorectal cancer (ATCC NO. HTB-37), and MCF7 breast cancer (ATCC NO. HTB-22) [34]. The acquired cell lines came from the American Type Culture Collection (Manassas, VA, USA). Invitrogen/Life Technologies, USA’s DMEM was utilised to cultivate the cells, together with 10% FBS (Hyclone, USA), 10 µg ml − 1 insulin (Sigma-Aldrich, Germany), and 1% penicillin-streptomycin. Cells were grown in 96-well plates prior to investigation, with a volume of 100 µl per well for each complete growth medium and the item under examination, and a density of 1.2–1.8 × 104 cells per well. A full day was spent with the cells. The MTT solution was reconstituted using three millilitres of the medium or a balanced salt solution free of serum or phenol red. Additionally, 10% of the culture medium’s volume was added to it. Depending on the type of cell and the maximum cell density, cultures were then incubated for two to four hours (two hours was generally adequate, but it was extended for low cell densities or cells demonstrating poor metabolic potential). After the incubation period, formazan crystals were dissolved by adding DMSO in a volume equivalent to the initial culture media. The absorbance of every plate was then determined by spectrophotometry using an ELISA plate reader (Model 550, Bio-Rad, USA) set to 570 nm. Three distinct experiments were carried out. GraphPad Prism 5 (Version 5.01, GraphPad Software, San Diego, CA, USA) was used to calculate IC50 values, or the concentration at which 50% of cell growth is inhibited.

### Metabolomics analysis

An Acquity Ultra Performance Liquid Chromatography system and a Synapt G2 HDMS quadrupole time-of-flight hybrid mass spectrometer were used to perform metabolomics profiling on the crude extract of the fungal culture, as described by [35] (Supplementary file).

## Computational study

### Network pharmacology-based analysis

#### Screening of Cladosporium sp. UR3 extract related targets genes

Based on chemical similarities, pharmacophore models, and protein interactions, the target genes of compounds identified from *Cladosporium* sp. UR3 extract were found through searches conducted in the Traditional Chinese Medicine Systems Pharmacology Database and Analysis Platform (TCMSP) database (https://old.tcmsp-e.com/index.php) [36], the Comparative Toxicogenomics Database (CTD) (http://ctdbase.org/) [37], and the Swiss Target Prediction Database (http://www.swisstargetprediction.ch/). After that, these target genes were translated into their corresponding gene names using the UniProt database (https://www.uniprot.org/) [38].

### Screening of human colorectal adenocarcinoma related target genes

Genes associated with human colorectal adenocarcinoma were obtained from the Cancer Cell Line Encyclopaedia (CCLE) database by using the keywords “human colorectal adenocarcinoma, colon carcinoma, and colon cancer” and the species limitation set to “Homo sapiens” [39]. The DisGeNET database (https://www.disgenet.org/) and the Comparative Toxicogenomic Database (https://ctdbase.org/) databases. Based on interactivenn (http://www.interactivenn.net/) [40] intersections, proteins associated with the disease and those linked to components overlapped and were recognised as potential targets of these components in human colorectal adenocarcinoma after duplicate targets were eliminated.

### Protein–protein interaction (PPI) network construction

A target gene query list was used to generate STRING version 12.0 on a PPI network (https://string-db.org/) [41] which was then exported to the free molecular and genetic interaction network visualisation, modelling, and analysis software Cytoscape 3.10.1 (USA) [42]. The Cytohubba plug-in was then used to screen the top 10 important genes.

### Gene ontology and kyoto encyclopedia of genes and genomes pathway analyses

We performed KEGG (Kyoto Encyclopaedia of Genes) enrichment analyses [43] for each putative target using the KEGG website (https://www.genome.jp/kegg), and we examined biological processes, cellular components, and related pathways using the Gene Ontology database (http://bioinformatics.sdstate.edu/go/) [44].

### Molecular docking

The Protein Data Bank [45], which provides the crystal structures of nine potential target genes, such as AKT1 (PDB ID: 4EJN), STAT3 (PDB ID: 6NJS), EGFR (PDB ID: 1M17), and ESR-1 (PDB ID: 1A52), was utilised [46]. To create the input files for the identified chemical compounds, protein structures, and cocrystallized ligands, AutoDockTools [47] was utilised. AutoDock Vina [48] was utilised to molecularly dock the proteins that were the subject of the study to chemical substances. The sizes of the grid boxes were limited to 27,000–3, and their “exhaustiveness” was set to 32, which is the ideal value for working with small boxes. To validate the docking procedure, co-crystallized ligands were redocked for protein crystal structures in complexes with binding molecules. The Discovery Studio Visualizer 17.2.0 was used to study and assess the docked drugs and important proteins.

## Results and discussion

### Phylogenetic identification

Strain UR3 was assigned to the genus *Cladosporium*. It shared highest partial 18 S rRNA gene sequences (99.6% and 99.5%) with *Cladosporium sphaerospermum* CBS 193.54 (NG_062724.1) and *Cladosporium sphaerospermum* CBS 193.54 (NG_062720.1). In addition strain UR3 shared the identical ITS region sequence with *C. sphaerospermum* CBS 193.54 (NR_111222.1). The phylogenetic placement to next related strains (type material of fungal species) is further illustrated in respective phylogenetic trees (Figure S1 and S2).

### Cytotoxic activity

The in vitro cytotoxic activity of the isolated fungal strain was examined against three cancer lines: colorectal carcinoma, breast cancer, and hepatocellular carcinoma (CACO2, MCF7, HEPG2), respectively. The ethyl acetate extract of *Cladosporium* sp. UR3 showed the highest potential against colon cancer followed by breast cancer, and hepatocellular carcinoma with IC_50_ values of: 4.7 ± 0.09, 7.2 ± 0.12, and 9.3 ± 0.18, respectively (Figure S3).

### Metabolomics profiling of the culture extract

**Fig. 1:**
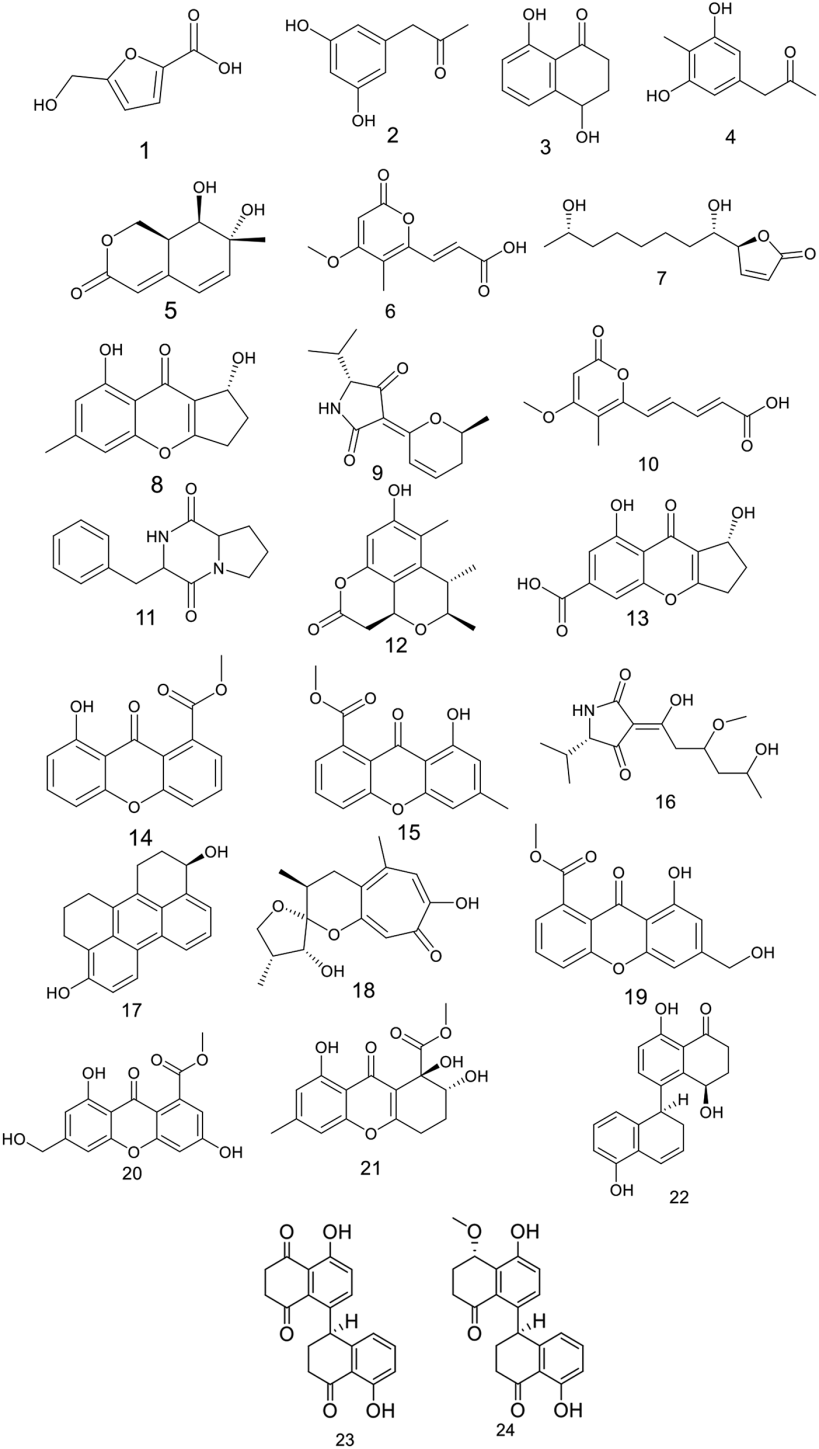
Dereplicated metabolites from the ethyl acetate extract of *Cladosporium* sp. UR3

Metabolomics is considered as an important tool to explore the chemical profile of each organism. Moreover, it aids in the discovery of new bioactive molecules and prevent the re-isolation of the known ones. It helps to enhance the techniques used during fungal fermentation and control the isolation of certain molecules [49]. Metabolomics analysis of the ethyl acetate extract of *Cladosporium* sp. UR3 using LC–HR–ESI–MS for dereplication purposes has resulted in the identification of a range of varied secondary metabolites that were dominated by phenolics, pyranones, tetramic acid derivatives, xanthones (Fig. 1, S4, S5, table S1). The detected compounds were identified by coupling MZmine with some databases, namely Marinlit and DNP. In view of that, the mass ion peak at *m/z* 141.0185 for the suggested molecular formula C_6_H_6_O_4_ was identified as Sumiki’s acid (**1**) which was previously isolated from *C. herbarum* [50]. Another mass ion peaks at *m/z* 165.0545, 177.0549, and 179.071, for the molecular formula C_9_H_10_O_3_, C_10_H_10_O_3_, C_10_H_12_O_3_, respectively, were identified as α-Acetylorcinol **(2)**, 4,8-Dihydroxy-1-tetralone **(3)**, 1-(3,5-Dihydroxy-4-methylphenyl)propan-2-one **(4)**, respectively. These metabolites were previously obtained from *C. perangustm* [51]. Whereas that at *m/z* 195.0659 for the molecular formula C_10_H_12_O_4_ was identified as Cladosporactone A **(5)** that was previously isolated from *C. cladosporioides* [52]. Additionally, the mass ion peak at *m/z* 209.0446, corresponding to the proposed molecular formula C_10_H_10_O_5_ was identified as Herbarin B **(6)**. It was formerly isolated from *C. herbarum* [53]. Iso-Cladospolide B **(7)** was dereplicated at *m/z* 229.1449 for the suggested molecular formula C_12_H_20_O_4_, previously obtained from *C. herbarum* [50]. Moreover, the mass ion peak at *m/z* 233.0727 and the predicted molecular formula C_13_H_12_O_4_ was identified as Coniochaetone B **(8)**, which was previously isolated from *C. halotolerans* [54]. The mass ion peak at *m/z* 234.1128 and the molecular formula C_13_H_17_NO_3_ was identified as Cladosporiumin I (**9**), which was previously obtained from *C. spherospermum* [55]. On the other hand, the mass ion peaks at *m/z* 235.0603, 245.1143, and 249.1126 in accordance with the molecular formulas C_12_H_12_O_5_, C_14_H_16_N_2_O_2_, and C_14_H_16_O_4_ were recognized as Herbarin A, (3*R*,8a*R*)-*Cyclo*(phenylalanylprolyl), Cladosporin C (**10**,** 11**,** 12**). Those compounds were previously isolated from *C. herbarum*,* C. cladosporioides*,* Cladosporium* sp., respectively [53]. Furthermore, the mass ion peak at *m/z* 261.040 and the suggested molecular formula C_13_H_10_O_6_ was identified as Coniochaetone K (**13**), which was earlier isolated from *C. halotolerans* [54].

Likewise, the mass ion peaks at *m/z* 269.0486, 283.0715 and the suggested molecular formula C_15_H_10_O_5_ and C_16_H_12_O_5_ were identified as Vertixanthone and Methyl 8-hydroxy-6-methyl-9-oxo-9*H*-xanthene-1-carboxylate (**14**,** 15**), respectively, which were previously isolated from *C. halotolerans* [54]. Cladosporiumin H (**16**) was dereplicated at *m/z* 286.1526, which in agreement with the molecular formula C_14_H_23_NO_5_. This compound was previously obtained from *Cladosporium* sp. [56]. The mass ion peak at *m/z* 291.1247 with the molecular formula C_20_H_18_O_2_ was identified as altertoxin IX (**17**) which was formerly obtained from *Cladosporium* sp. [57].

Furthermore, Malettinin B (**18**) was dereplicated at *m/z* 293.1297 and in accordance with the molecular formula C_16_H_20_O_5_, which was previously isolated from *Cladosporium* sp. [58]. Likewise, the mass ion peaks at *m/z* 299.0592, 315.0539, 321.0882 and the molecular formula C_16_H_12_O_6_, C_16_H_12_O_7,_ C_16_H_16_O_7_ were identified as Methyl 8-hydroxy-6-(hydroxymethyl)-9-oxo-9*H*-xanthene-1-carboxylate, Conioxanthone A, α-Diversonolic ester (**19**,** 20**,** 21**), respectively, which were previously isolated from *C. halotolerans* [54]. Additionally, the mass ion peak at *m/z* 321.1241 in keeping with the molecular formula C_20_H_18_O_4_, was dereplicated as altertoxin XII **(22)**, which was formerly purified from *Cladosporium* sp. [57]. Cladosporol H, F (**23**,** 24**) were characterized at *m/z* 337.0983, 351.1286 and the molecular formula C_20_H_16_O_5,_ C_21_H_20_O_5_, respectively. These compounds were previously isolated from *C. cladosporioides* [59].

### Computational study

#### Network pharmacology-based analysis

Based on the remarkable cytotoxic activity demonstrated by the *Cladosporium* extract on the human colorectal adenocarcinoma cell line (CACO2), we recognize the utmost significance of constructing a gene network for human colorectal adenocarcinoma. This effort is critical for advancing our comprehension of the genetic components involved in the disease and for formulating personalized treatment strategies.

### Screening of Cladosporium sp. UR3 extract related targets genes

A total of 430 target genes associated with twenty-four compounds dereplicated from *Cladosporium* sp. UR3 were gathered through data sources such as TCMSP, CTD, and Swiss Target Prediction. These genes were then converted into their canonical gene names utilizing the UniProt database.

#### Screening of human colorectal adenocarcinoma related target genes

**Fig. 2:**
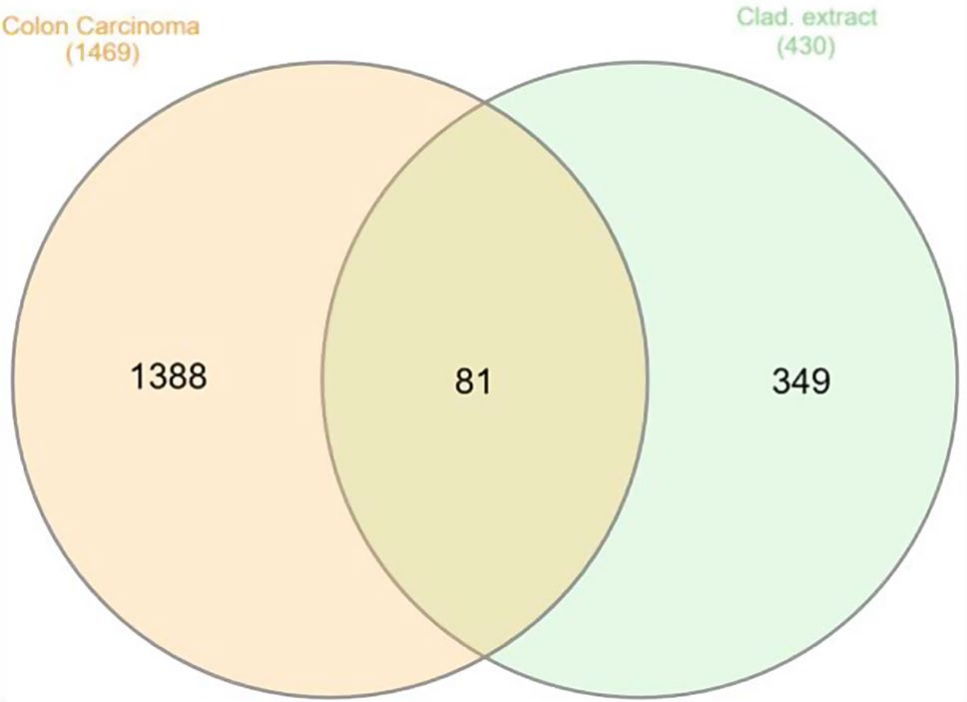
Venn diagram for the integrated analysis of the related targets of *Cladosporium* sp. UR3 dereplicated compounds and colorectal carcinoma

A total of 1,469 common target genes, recognized for their relevance to human colorectal adenocarcinoma, were meticulously gathered from CCLE, CTD, and DisGeNET databases. These genes were extracted using specific search criteria, including the keywords " human colorectal adenocarcinoma, colon carcinoma and colon cancer " with a species restriction limited to “Homo sapiens.” After eliminating any duplicate entries, a Venn diagram was meticulously crafted to visualize the overlap between the target genes influenced by compounds dereplicated from *Cladosporium* sp. UR3 and the potential target genes associated with human colorectal adenocarcinoma. This visual representation revealed a total of 81 shared targets, as illustrated in (Fig. 2).

### Protein–protein Interaction (PPI) Network Construction

**Fig. 3:**
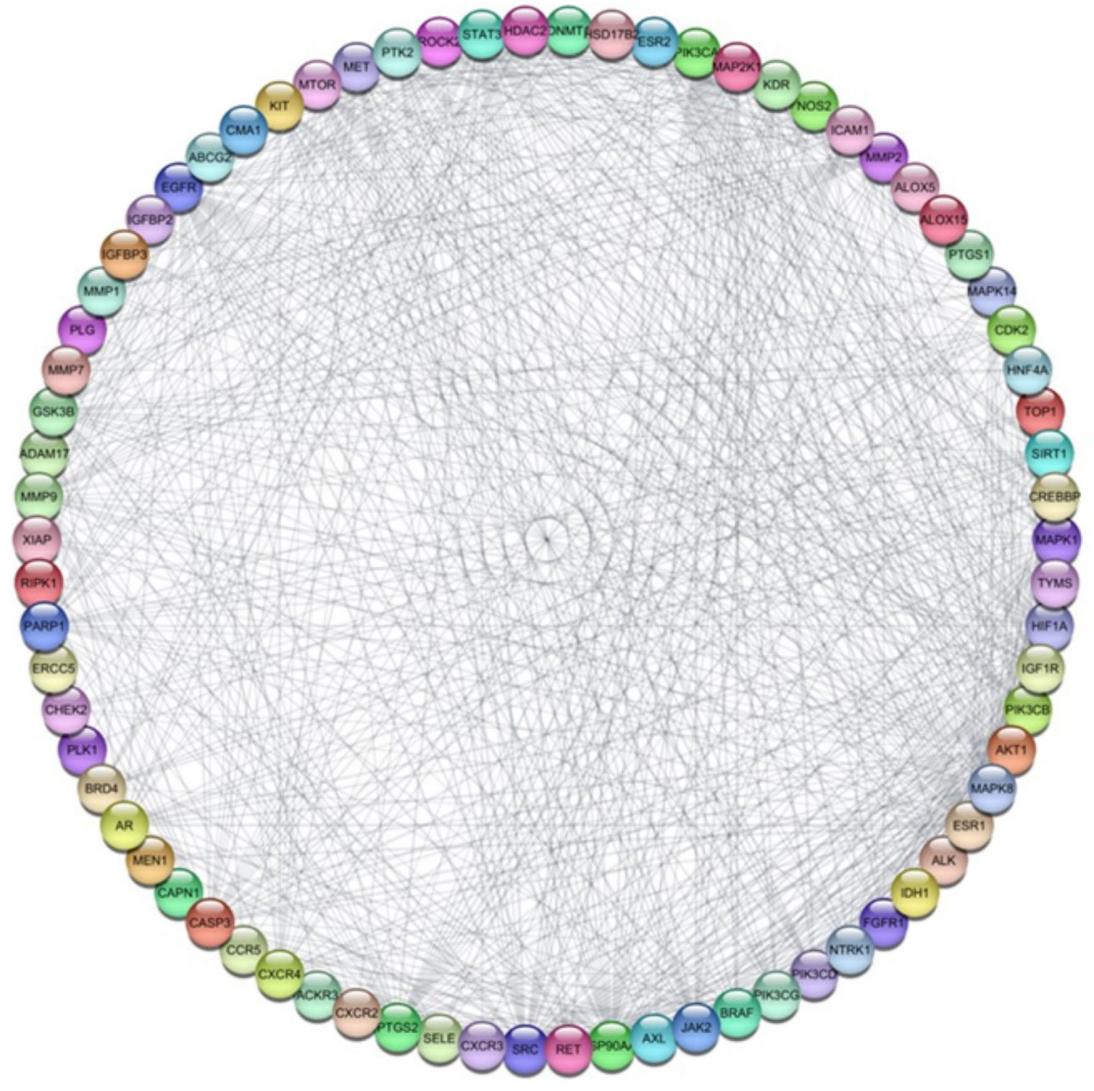
Network nodes represent 77 protein targets, and the edges represent protein–protein interactions

**Fig. 4:**
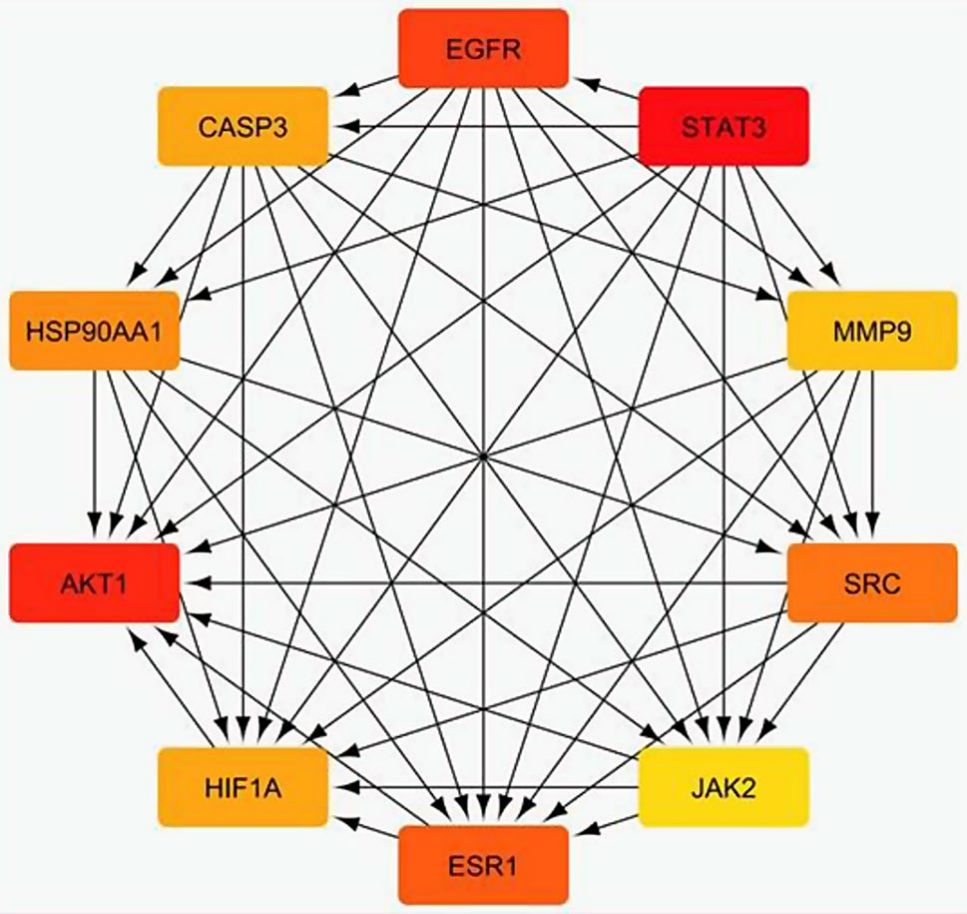
Network nodes represent the top 10 hub genes: the darker the color, the higher the score and the stronger the connection

The 81 common target genes were inputted into the STRING database to conduct an analysis of protein-protein interactions (PPI). The outcomes were then utilized to create a PPI network diagram using Cytoscape 3.10.1 software. This network consisted of 77 nodes (after excluding four nodes that were not connected) and 746 edges, with an average node connectivity of 19.26, as illustrated in Fig. 3. Subsequently, the Cytohubba plugin was employed to identify and extract the top ten significant genes based on their degree of connectivity within the network, as shown in Fig. 4. The greater the degree value of any gene, the more evident it will be in disease pathogenesis. These genes are STAT3, AKT1, EGFR, ESR1, SRC, HSP90AA1, CASP3, HIF1A, MMP9, and JAK2. The topological parameters, including node degree, betweenness, and closeness for each protein, were then summarized in Table 1.

**Table 1:** Topological parameters of top 10 hub genes

No.	Name	Target	Degree	Betweenness	Closeness
1	Signal transducer and activator of transcription 3	STAT3	56	0.1069	0.7700
2	RAC-alpha serine/threonine-protein kinase	AKT1	54	0.0836	0.7549
3	Epidermal growth Factor	EGFR	53	0.0995	0.7624
4	Estrogen receptor alpha	ESR1	47	0.0591	0.7064
5	Proto-oncogene tyrosine-protein kinase	SRC	45	0.0338	0.6937
6	Heat shock protein HSP 90-alpha	HSP90AA1	43	0.0281	0.6754
7	Caspase-3	CASP3	42	0.0431	0.6754
8	Hypoxia-inducible factor 1-alpha	HIF1A	42	0.0438	0.6875
9	Matrix metalloproteinase-9	MMP9	39	0.0341	0.6525
10	Tyrosine-protein kinase JAK2	JAK2	35	0.0137	0.6210

#### Gene ontology and kyoto encyclopedia of genes and genomes pathway analyses

To construct a network that links target genes with specific pathways, we created this network by connecting all the identified genes influenced by *Cladosporium* sp. UR3 dereplicated compounds and their interactions with colorectal carcinoma, using information from the KEGG database and ShinyGO database.

**Fig. 5:**
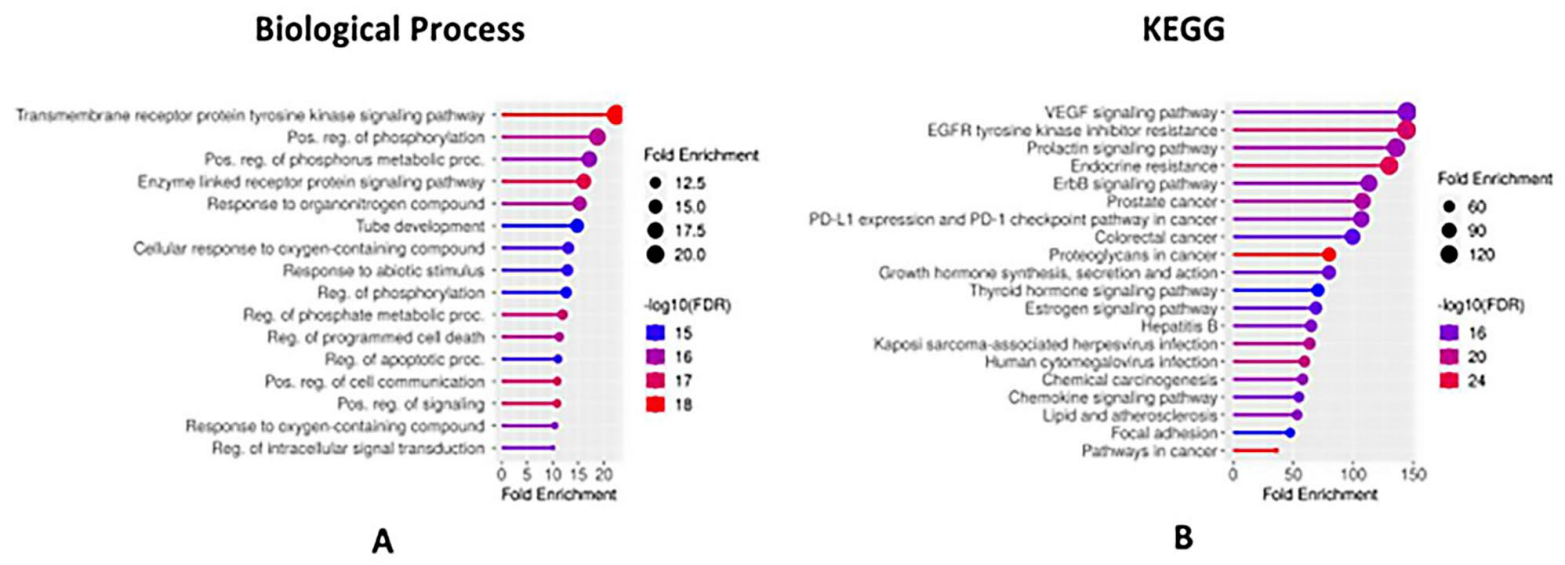
(**A**) Functional enrichment analysis of filtered 24 protein-coding genes by ShinyGO (Biological process); (**B**) KEGG pathway analysis

The Gene Ontology (GO) functional analysis of selected genes suggested that transmembrane receptor protein tyrosine kinase signaling pathway, positive regulation of phosphorylation and positive regulation of phosphorous metabolic process are among the top pathways in the biological process category (Fig. 5A) correlated with colorectal cancer. Subsequently, we performed a Kyoto Encyclopedia of Genes and Genomes (KEGG) pathway analysis to pinpoint the significant signaling pathways linked to colorectal cancer. The KEGG analysis of the selected protein-coding genes revealed potential involvement in cancer through pathways including the VEGF signaling pathway, EGFR tyrosine kinase resistance, and the prolactin signaling pathway, as depicted in (Fig. 5B).

#### Molecular docking

Finally, docking studies were conducted to explore potential targets for those compounds that were identified within the extract. In our study, twenty-four annotated compounds were subjected to top four hub genes, STAT3 (pdb: 6NJS), AKT-1 (pdb: 4EJN), EGFR (pdb: 1M17) and ESR-1(pdb: 1A52), active sites. The docking scores of these compounds against these four target proteins are presented in Table 2. To validate the docking protocol, we also performed re-docking of co-crystallized ligands, ensuring that the RMSD between the crystal pose and the docked pose was less than 2 Å for a successful validation. In this context, we compared the docking score of the co-crystallized ligand with those of the tested compounds. Generally, several of the tested compounds displayed better docking scores especially in RAC-alpha serine/threonine-protein kinase (AKT-1), Epidermal growth factor (EGFR) and Estrogen receptor alpha (ESR-1) targets.

**Fig. 6:**
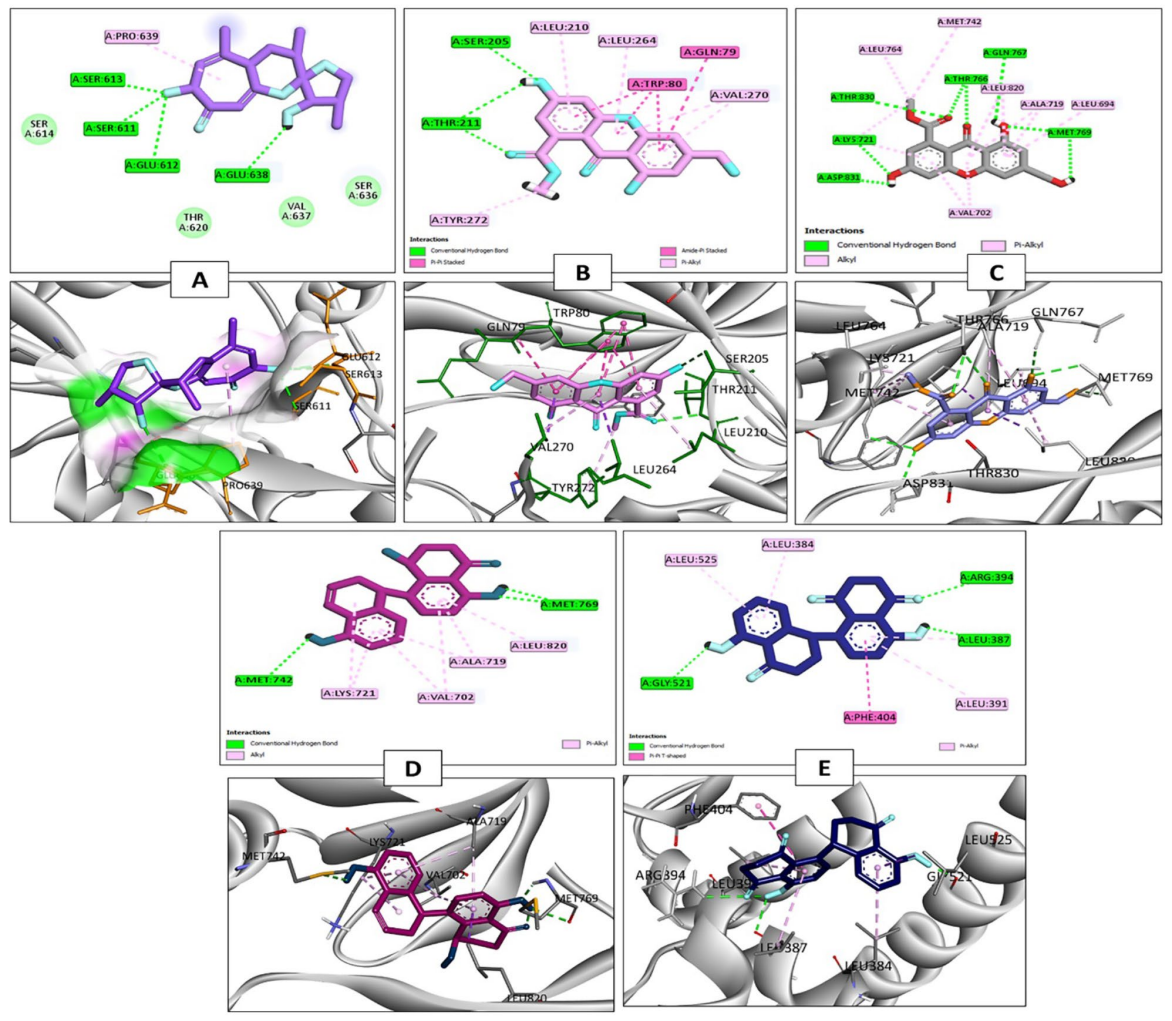
2D and 3D interaction diagrams of (**A**) Compound 18 in STAT3 active site (PBD ID 6NJS), (**B**) Compound 20 in AKT-1 active site (PDB ID 4EJN), (**C**) compound 20 in EGFR active site (PDB ID 1M17), (**D**) Compound 22 in EGFR active site (PDB ID 1M17), (**E**) Compound 23 in ESR-1 active site (PDB ID 1A52)

Upon docking, compound 18 within the active site of STAT3 showed good docking score = -5.36 kal/mol through formation of four hydrogen bond connections with the amino acid residues Ser 611, Glu 612, Ser 613, and Glu 638. Additionally, a hydrophobic interaction was established with the amino acid residue Pro 639 (as shown in Fig. 6A).

Among docked compounds, the interaction of compound **20** against AKT-1 active site showed the least docking score of -8.58 kcal/mol. This remarkable interaction was characterized by the establishment of three hydrogen bond interactions with Ser 205 and Thr 211 amino acid residues alongside a series of hydrophobic interactions involving key amino acid residues, namely Gln 79, Trp 80, Leu 210, Leu 264, Val 270, and Tyr 272 (Fig. 6B**)**.

When docking with the EGFR tyrosine kinase active site, both compounds **20** and **22** exhibited the most favorable S score values, with values of -8.46 and − 8.50 kcal/mol, respectively. Compound **20** established several hydrogen bond interactions, notably with the critical amino acid residue Met 769, which plays a crucial role in inhibiting EGFR activity, as illustrated in Fig. 6C. Furthermore, compound **20** also demonstrated hydrophobic interactions. On the other hand, compound **22** formed three hydrogen bond interactions within the EGFR active site, with two of these interactions involving the amino acid residue Met 769 and another involving the amino acid residue Met 742. Additionally, compound **22** engaged in hydrophobic interactions with amino acid residues Val 702, Ala 719, Lys 721, and Leu 820 (Fig. 6D**)**.

When the dereplicated compounds were docked into the ESR-1 active site, it was observed that compound **23** displayed the lowest binding energy score, which was calculated at -8.02 kcal/mol. This interaction was characterized by the formation of three hydrogen bond interactions with the amino acid residues Leu 387, Arg 394, and Gly 521, along with engaging in hydrophobic interactions with the amino acid residues Leu 384, Leu 391, Phe 404, and Leu 525 (Fig. 6E**)**.

**Table 2:** Docking scores of tested compounds

Compound No.	Docking Score (kcal/mol)			
	Colorectal Carcinoma targets			
	STAT3 (6NJS)	AKT-1 (4EJN)	EGFR (1M17)	ESR-1 (1A52)
**1**	-4.34	-4.86	-4.94	-4.88
**2**	-4.34	-6.63	-6.22	-6.52
**3**	-4.6	-6.64	-6.49	-7.19
**4**	-4.4	-7.02	-7.03	-7.34
**5**	-4.47	-5.95	-6.62	-6.56
**6**	-3.81	-6.68	-4.37	ND
**7**	-4.1	-3.45	-3.95	-3.59
**8**	-3.61	-6.48	-7.68	-7.95
**9**	ND	-6.74	-7.51	ND
**10**	-4.55	-5.77	-5	ND
**11**	-4.15	-7.65	-7.57	-7.07
**12**	-2.33	-6.78	-6.68	ND
**13**	-4.26	-6.3	-7.43	ND
**14**	-4.96	-7.48	-5.75	-7.42
**15**	-4.84	-7.56	-6.2	ND
**16**	-4.33	-6.43	-7.24	-6.4
**17**	-3.65	-8.33	-6.98	ND
**18**	-5.36	-6.45	-7.35	-7.91
**19**	-4.62	-8.35	-5.88	ND
**20**	-4.85	-8.58	-8.46	ND
**21**	-4.68	-6.57	-7.48	ND
**22**	-4.08	-5.73	-8.5	ND
**23**	-3.92	-6.8	-6.44	-8.02
**24**	-4.39	-6.45	-6.22	ND
**Co-crystalized Ligand**	-10.01	-14.46	-9.11	-11.15

ND: Not Detected

Finally, we conclude that our study suggests the investigated metabolites of *Cladosporium* sp. UR3 might have activity against colorectal carcinoma, especially compounds 20, 22, and 23 targeting AKT-1, EGFR, and ESR-1. This rationale is based on the known crucial roles of these proteins in colorectal carcinoma development and progression.

## Conclusion

Our current study described the isolation and purification of a fungal isolate from the Red Sea sponge, *Hyrtios sp.*, which was molecularly identified as *Cladosporium* sp. UR3. This endophytic fungus has been shown to be a promising resource of natural metabolites particularly when it was fermented using SDA culture media. Additionally, investigating the in vitro cytotoxic potential of the crude extract of *Cladosporium* sp. UR3 revealed its powerful activity against colorectal, breast and hepatocellular carcinoma cell lines, with IC_50_ values of 4.7 ± 0.09, 7.2 ± 0.12, and 9.3 ± 0.18 µg/mL, respectively. Interestingly, metabolomics profiling of the sponge-associated fungi lead to the characterization of 24 metabolites described as phenolics, pyranones, tetramic acid derivatives, and xanthones. Moreover, molecular docking analysis of the dereplicated secondary metabolites suggested their AKT-1, ESR-1, and EGFR tyrosine kinase inhibitory potential as a plausible mechanism for their cytotoxic activities. These findings highlighted the significance of marine fungi as an important reservoir of bioactive molecules for the discovery of cytotoxic medications with natural origin.

## Electronic supplementary material

Below is the link to the electronic supplementary material.

Supplementary Material 1

## Data Availability

“All data generated or analyzed during this study are included in this article (and its supplementary information files). The ITS region and the small subunit ribosomal RNA gene were individually uploaded to GenBank and assigned Accession Numbers OR900633.1 and OR900731.1.”

## References

[1] Cheng Z, Li M, Dey R, Chen, YJJoh, editors. oncology. Nanomaterials for cancer therapy: Current progress and perspectives. 2021;14(1):1–27. 10.1186/s13045-021-01096-0 PMC8165984 34059100

[2] Mantovani A, Allavena P, Marchesi F, Garlanda CJ, NRDD. Macrophages as tools and targets in cancer therapy. 2022;21(11):799–820. 10.1038/s41573-022-00520-5 PMC9380983 35974096

[3] Klein APJ, NrGhepatology. Pancreatic cancer epidemiology: understanding the role of lifestyle and inherited risk factors. 2021;18(7):493–502. 10.1038/s41575-021-00457-x PMC9265847 34002083

[4] Mattiuzzi C. Lippi GJJoe, health g. Curr cancer Epidemiol. 2019;9(4):217. 10.2991/jegh.k.191008.001 PMC7310786 31854162

[5] Liu Y-Q, Wang X-L, He D-H, Cheng Y-XJ, Phytomedicine. Protection against chemotherapy-and radiotherapy-induced side effects: a review based on the mechanisms and therapeutic opportunities of phytochemicals. 2021;80:153402. 10.1016/j.phymed.2020.153402 33203590

[6] Huang M, Lu J-J, Ding JJNp bioprospecting. Nat Prod cancer Therapy: Past Present Future. 2021;11:5–13. 10.1007/s13659-020-00293-7 PMC7933288 33389713

[7] Vuong TVJA. Natural products and their derivatives with antibacterial, antioxidant and anticancer activities. MDPI; 2021. p. 70. 10.3390/antibiotics10010070 PMC7828331 33450907

[8] Zou J-Y, Chen Q-L, Luo X-C, Damdinjav D, Abdelmohsen UR, Li H-Y, et al. Nat Prod Reverse cancer Multidrug Resist. 2024;15:1348076. 10.3389/fphar.2024.1348076 PMC10988293 38572428

[9] Said AAE, Mahmoud BK, Attia EZ, Abdelmohsen UR, Fouad MAJR. Bioactive Nat Prod Mar Sponges Belonging Family Hymedesmiidae. 2021;11(27):16179–91. 10.1039/d1ra00228g PMC9031984 35479127

[10] Hisham Shady N, Zhang J, Khalid Sobhy S, Hisham M, Glaeser SP, Alsenani F, et al., NPR. Metabolomic profiling and cytotoxic potential of three endophytic fungi of the genera aspergillus, Penicillium and Fusarium isolated from Nigella sativa seeds assisted with docking studies. 2023;37(17):2905–10. 10.1080/14786419.2022.2136660 36305731

[11] Singh YD, Jena B, Ningthoujam R, Panda S, Priyadarsini P, Pattanayak S et al. Potential bioactive molecules from natural products to combat against coronavirus. 2020:1–12.

[12] Khalifa SA, Elias N, Farag MA, Chen L, Saeed A, Hegazy M-EF, et al., MD. Marine natural products: a source of novel anticancer drugs. 2019;17(9):491. 10.3390/md17090491 PMC6780632 31443597

[13] Abu-Izneid T, Rauf A, Shariati MA, Khalil AA, Imran M, Rebezov M, et al. Sesquiterpenes and their derivatives-natural anticancer compounds. Update. 2020;161:105165. 10.1016/j.phrs.2020.105165 32835868

[14] Ashraf MAJBri. Phytochemicals as potential anticancer drugs: time to ponder nature’s bounty. 2020;2020. 10.1155/2020/8602879 PMC7013350 32076618

[15] Rani D, Garg V, Dutt RJA-CAMC. Anticancer potential of Azole Containing Marine Natural products. Curr Future Perspect. 2021;21(15):1957–76. 10.2174/1871520621666210112112422 33438564

[16] Abdelmohsen UR, Bayer K, Hentschel U, JNpr. Diversity, abundance and natural products of marine sponge-associated actinomycetes. 2014;31(3):381–99. 10.1039/c3np70111e 24496105

[17] Shady NH, Abdelmohsen UR, AboulMagd AM, Amin MN, Ahmed S, Fouad MA, et al. Cytotoxic potential of the Red Sea sponge Amphimedon Sp. Supported silico modelling Dereplication Anal. 2021;35(24):6093–8. 10.1080/14786419.2020.1825430 32975127

[18] Abdelmohsen UR, Yang C, Horn H, Hajjar D, Ravasi T, Hentschel U, JMd. Actinomycetes from Red Sea sponges: sources for chemical and phylogenetic diversity. 2014;12(5):2771–89. 10.3390/md12052771 PMC4052315 24824024

[19] Ameen F, AlNadhari S, Al-Homaidan AAJSJBS. Mar Microorganisms as Untapped Source Bioactive Compd. 2021;28(1):224–31. 10.1016/j.sjbs.2020.09.052 PMC7783642 33424301

[20] Samirana PO, Jenie RI, Murti YB, Setyowati EPJ, JoAPS. Application of metabolomics on marine sponges and sponge-associated microorganisms: a review. 2022;12(7):018–33.

[21] Said Hassane C, Fouillaud M, Le Goff G, Sklirou AD, Boyer JB, Trougakos IP, et al. Microorganisms associated with the marine sponge Scopalina Hapalia: a reservoir of bioactive molecules to slow down the aging process. 2020;8(9):1262. 10.3390/microorganisms8091262 PMC7570120 32825344

[22] Noman E, Al-Shaibani MM, Bakhrebah MA, Almoheer R, Al-Sahari M, Al-Gheethi A, et al., JOF. Potential of anti-cancer activity of secondary metabolic products from marine fungi. 2021;7(6):436. 10.3390/jof7060436 PMC8229146 34070936

[23] Cao DT, Doan TMH, Pham VC, Le THM, Chae J-W, Yun H-y, et al. Mol Des Anticancer Drugs Mar fungi Derivatives. 2021;11(33):20173–9. 10.1039/d1ra01855h PMC9033662 35479875

[24] Ameen F, Al-Homaidan AA, Al-Sabri A, Almansob A, AlNAdhari S, JAN. Anti-oxidant, anti-fungal and cytotoxic effects of silver nanoparticles synthesized using marine fungus Cladosporium halotolerans. 2023;13(1):623–31.

[25] Mohamed GA, Ibrahim SR, JMD. Untapped potential of marine-associated Cladosporium species: an overview on secondary metabolites, biotechnological relevance, and biological activities. 2021;19(11):645. 10.3390/md19110645 PMC8622643 34822516

[26] Salvatore MM, Andolfi A, Nicoletti R, JM. The genus Cladosporium: a rich source of diverse and bioactive natural compounds. 2021;26(13):3959. 10.3390/molecules26133959 PMC8271404 34203561

[27] Abdelwahab MF, Fouad MA, Kamel MS, Özkaya FC, Kalscheuer R, Müller WE, et al. Tanzawaic acid derivatives from freshwater sediment-derived fungus Penicillium Sp. Fitoterapia. 2018;128:258–64. 29778575 10.1016/j.fitote.2018.05.019

[28] Manilal A, Sabarathnam B, Kiran G, Sujith S, Shakir C, Selvin J. Antagonistic potentials of marine sponge associated fungi Aspergillus Clavatus MFD15. Asian J Med Sci. 2010;2(4):195–200.

[29] Fliegerová K, Mrazek J, Voigt, KJFm. Differentiation of anaerobic polycentric fungi by rDNA. PCR-RFLP. 2006;51:273–7. 10.1007/BF02931811 17007423

[30] Tamura K, Stecher G. Kumar S, JMb, evolution. MEGA11: molecular evolutionary genetics analysis version 11. 2021;38(7):3022–7. 10.1093/molbev/msab120 PMC8233496 33892491

[31] Kimura, MJ, Jome. A simple method for estimating evolutionary rates of base substitutions through comparative studies of nucleotide sequences. 1980;16:111–20. 10.1007/BF01731581 7463489

[32] Katoch M, Phull S, Vaid S, Singh S, JBm. Diversity, phylogeny, anticancer and antimicrobial potential of fungal endophytes associated with Monarda citriodora L. 2017;17:1–13. 10.1186/s12866-017-0961-2 PMC5339955 28264654

[33] Ahmed AM, Ibrahim AM, Yahia R, Shady NH, Mahmoud BK, Abdelmohsen UR, et al., JBm. Evaluation of the anti-infective potential of the seed endophytic fungi of Corchorus olitorius through metabolomics and molecular docking approach. 2023;23(1):355. 10.1186/s12866-023-03092-5 PMC10656998 37980505

[34] Rasheed T, Bilal M, Iqbal HM, Li CJC, Biointerfaces SB. Green biosynthesis of silver nanoparticles using leaves extract of Artemisia vulgaris and their potential biomedical applications. 2017;158:408–15. 10.1016/j.colsurfb.2017.07.020 28719862

[35] Osama N, Bakeer W, Raslan M, Soliman HA, Abdelmohsen UR, Sebak M, JRSos. Anti-cancer and antimicrobial potential of five soil streptomycetes: a metabolomics-based study. 2022;9(2):211509. 10.1098/rsos.211509 PMC8825997 35154794

[36] Ru J, Li P, Wang J, Zhou W, Li B, Huang C, et al. TCMSP: a database of systems pharmacology for drug discovery from herbal medicines. J Cheminform. 2014;6:13. 24735618 10.1186/1758-2946-6-13 PMC4001360

[37] Davis AP, Wiegers TC, Wiegers J, Wyatt B, Johnson RJ, Sciaky D, et al. CTD tetramers: a new online tool that computationally links curated chemicals, genes, phenotypes, and diseases to inform molecular mechanisms for environmental health. Toxicol Sci. 2023;195(2):155–68. 37486259 10.1093/toxsci/kfad069 PMC10535784

[38] UniProt C. UniProt: the universal protein knowledgebase in 2021. Nucleic Acids Res. 2021;49(D1):D480–9. 33237286 10.1093/nar/gkaa1100 PMC7778908

[39] Ghandi M, Huang FW, Jane-Valbuena J, Kryukov GV, Lo CC, McDonald ER 3, et al. Next-generation characterization of the Cancer Cell Line Encyclopedia. Nature. 2019;569(7757):503–8. 31068700 10.1038/s41586-019-1186-3 PMC6697103

[40] Heberle H, Meirelles GV, da Silva FR, Telles GP, Minghim R. InteractiVenn: a web-based tool for the analysis of sets through Venn diagrams. BMC Bioinformatics. 2015;16(1):169. 25994840 10.1186/s12859-015-0611-3 PMC4455604

[41] Szklarczyk D, Kirsch R, Koutrouli M, Nastou K, Mehryary F, Hachilif R, et al. The STRING database in 2023: protein-protein association networks and functional enrichment analyses for any sequenced genome of interest. Nucleic Acids Res. 2023;51(D1):D638–46. 36370105 10.1093/nar/gkac1000 PMC9825434

[42] Shannon P, Markiel A, Ozier O, Baliga NS, Wang JT, Ramage D, et al. Cytoscape: a software environment for integrated models of biomolecular interaction networks. Genome Res. 2003;13(11):2498–504. 14597658 10.1101/gr.1239303 PMC403769

[43] Kanehisa M, Furumichi M, Sato Y, Ishiguro-Watanabe M, Tanabe M. KEGG: integrating viruses and cellular organisms. Nucleic Acids Res. 2021;49(D1):D545–51. 33125081 10.1093/nar/gkaa970 PMC7779016

[44] Ge SX, Jung D, Yao R. ShinyGO: a graphical gene-set enrichment tool for animals and plants. Bioinformatics. 2020;36(8):2628–9. 31882993 10.1093/bioinformatics/btz931 PMC7178415

[45] Berman HM, Westbrook J, Feng Z, Gilliland G, Bhat TN, Weissig H, et al. The Protein Data Bank. Nucleic Acids Res. 2000;28(1):235–42. 10592235 10.1093/nar/28.1.235 PMC102472

[46] Morris GM, Huey R, Lindstrom W, Sanner MF, Belew RK, Goodsell DS, et al. AutoDock4 and AutoDockTools4: automated docking with selective receptor flexibility. J Comput Chem. 2009;30(16):2785–91. 19399780 10.1002/jcc.21256 PMC2760638

[47] Trott O, Olson AJ. AutoDock Vina: improving the speed and accuracy of docking with a new scoring function, efficient optimization, and multithreading. J Comput Chem. 2010;31(2):455–61. 19499576 10.1002/jcc.21334 PMC3041641

[48] Jaghoori MM, Bleijlevens B, Olabarriaga SD. 1001 ways to run AutoDock Vina for virtual screening. J Comput Aided Mol Des. 2016;30(3):237–49. 26897747 10.1007/s10822-016-9900-9 PMC4801993

[49] Tawfike AF, Tate R, Abbott G, Young L, Viegelmann C, Schumacher M, et al. Metabolomic tools to assess the chemistry and bioactivity of endophytic aspergillus strain. Chem Biodivers. 2017;14(10):e1700040. 10.1002/cbdv.201700040 28672096

[50] Jadulco R, Proksch P, Wray V, Sudarsono, Berg A, Gräfe UJ, Jonp. New macrolides and furan carboxylic acid derivative from the sponge-derived Fungus Cladosporium h erbarum. 2001;64(4):527–30. 10.1021/np000401s 11325242

[51] Fan Z, Sun Z-H, Liu H-X, Chen Y-C, Li H-H, Zhang W-MJ, JoAnpr. Perangustols A and B, a pair of new azaphilone epimers from a marine sediment-derived fungus Cladosporium perangustm FS62. 2016;18(11):1024–9. 10.1080/10286020.2016.1181623 27240037

[52] He ZH, Zhang G, Yan QX, Zou ZB, Xiao HX, Xie CL, et al. Ch & Bio. Cladosporactone A, a unique polyketide with 7-methylisochromen‐3‐one skeleton from the deep‐sea‐derived fungus Cladosporium cladosporioides. 2020;17(6):e2000158. 10.1002/cbdv.202000158 32259395

[53] Jadulco R, Brauers G, Edrada RA, Ebel R, Wray V, Sudarsono, et al., JNP. New metabolites from sponge-derived fungi curvularia l unata and cladosporium h erbarum. 2002;65(5):730–3. 10.1021/np010390i 12027752

[54] Wang C-N, Lu H-M, Gao C-H, Guo L, Zhan Z-Y, Wang J-J, et al., NPR. Cytotoxic benzopyranone and xanthone derivatives from a coral symbiotic fungus Cladosporium halotolerans GXIMD 02502. 2021;35(24):5596–603. 10.1080/14786419.2020.1799363 32713199

[55] Liang X, Huang Z-H, Ma X, Qi S-H, JMd. Unstable tetramic acid derivatives from the deep-sea-derived fungus Cladosporium sphaerospermum EIODSF 008. 2018;16(11):448. 10.3390/md16110448 PMC6266709 30445739

[56] Huang Z-h, Nong X-h, Liang X, Qi S-hJT. New tetramic acid derivatives from the deep-sea-derived fungus Cladosporium sp. SCSIO z0025. 2018;74(21):2620–6.

[57] Zhang F, Zhou L, Kong F, Ma Q, Xie Q, Li J et al., JMD. Altertoxins with quorum sensing inhibitory activities from the marine-derived fungus Cladosporium sp. KFD33. 2020;18(1):67. 10.3390/md18010067 PMC7024320 31963874

[58] Silber J, Ohlendorf B, Labes A, Wenzel-Storjohann A, Näther C, Imhoff JF, JFiMS. Malettinin E, an antibacterial and antifungal tropolone produced by a marine Cladosporium strain. 2014;1:35.

[59] Li H-L, Li X-M, Mándi A, Antus S, Li X, Zhang P, et al. Characterization of cladosporols from the marine algal-derived endophytic fungus Cladosporium cladosporioides EN-399 and configurational revision of the previously reported cladosporol derivatives. 2017;82(19):9946–54. 10.1021/acs.joc.7b01277 28853887

