# Supplementary material for: Hyrtios sp.-associated Cladosporium sp. UR3 as a potential source of antiproliferative metabolites
Source: BMC Microbiol. 2024 Nov 1;24:445. doi: 10.1186/s12866-024-03560-6 (PMC11529160; doi:10.1186/s12866-024-03560-6)
Supplement: Supplementary file 1 — Supplementary Material 1 [file 12866_2024_3560_MOESM1_ESM.docx]

**Supplementary data**

***Hyrtios* sp. associated *Cladosporium* sp. UR3 as a potential source of antiproliferative metabolites supported by docking and pharmacological network analyses**

Omnia Hesham Abdelhafez^a^, Abeer H. Elmaidomy^b^, Mohamed Hisham^c^, Stefanie P. Glaeser^d^, Peter Kämpfer^d^, Jun Wu^e^, Usama Ramadan Abdelmohsen^a,f*^

**^a^** Department of Pharmacognosy, Faculty of Pharmacy, Deraya University, New Minia City, Minia, Egypt

^b^ Department of Pharmacognosy, Faculty of Pharmacy, Beni-Suef University, Beni-Suef 62514, Egypt

^c^ Department of Pharmaceutical Chemistry, Faculty of Pharmacy, Deraya University, New-Minia 61512, Egypt

^d^ Institute of Applied Microbiology, Justus-Liebig University Gießen, Gießen, Germany

^e^ Guangdong Key Laboratory for Research and Development of Natural Drugs, College of Pharmacy, Guangdong Medical University, Dongguan 523808, China

^f^ Department of Pharmacognosy, Faculty of Pharmacy, Minia University, Minia 61519, Egypt

***** Correspondence: authors: usama.ramadan@mu.edu.eg (URA).

***Materials and Methods:***

***Metabolomics analysis***

Metabolomics profiling was carried out on the crude extract of *Cladosporium* sp. UR3 using an Acquity Ultra Performance Liquid Chromatography system coupled to a Synapt G2 HDMS quadrupole time-of-flight hybrid mass spectrometer (Waters, Milford, USA) ^(^[^1^](#_ENREF_1)^)^. Additionally, Ms converter software was utilized to convert the raw data into both positive and negative ionization files. After that, the obtained data were subjected to the data mining software MZmine 2.10 (Okinawa Institute of Science and Technology Graduate University, Japan) for deconvolution, peak picking, alignment, deisotoping, and formula prediction. The MarinLit ^(^[^2^](#_ENREF_2)^)^ database was finally used for identification of the detected compounds.


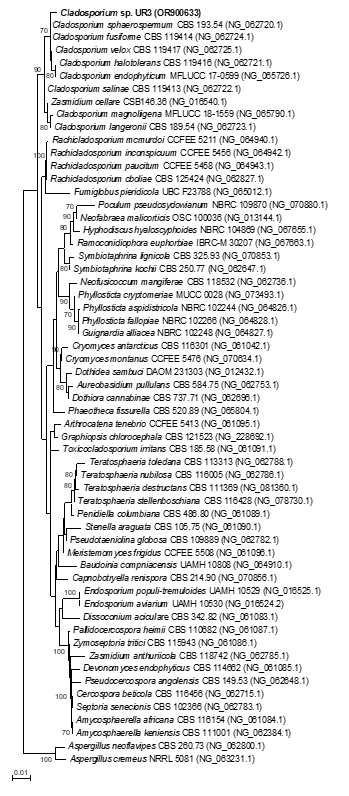


**Figure S1** Phylogenetic tree based on partial 18S rRNA gene sequences showing the phylogenetic placement of strain UR3 to next related fungal species. The tree was generated in MEGA11 using the maximum likelihood method and the Kimura-2-parameter model. Sequences of next related strains were selected based on the National Center for Biotechnology Information (NCBI) Nucleotide BLAST analysis and the rRNA/ITS NCBI RefSeq curated datasets for the respective DNA regions (Bioproject PRJNA224725; updated 2023-12-19). Numbers at nodes represent bootstrap values (>70%). This analysis involved 59 nucleotide sequences and a total of 1143 nucleotide positions in the final dataset. Sequences of two *Aspergillus* species were used as outgroup to root the tree. Scale bar: 0.01 nucleotide substitutions per sequence position.


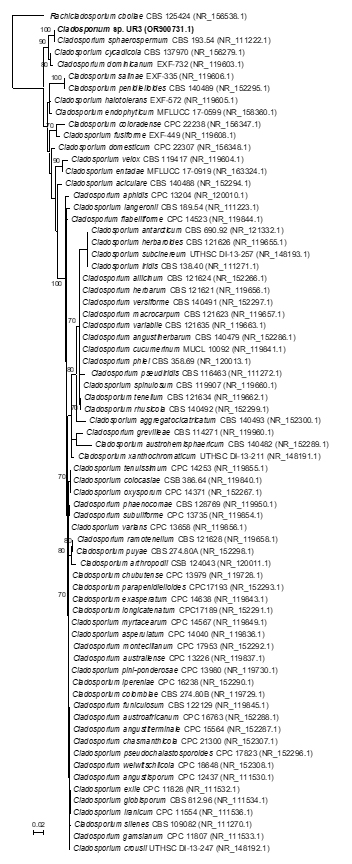


**Figure S2** Phylogenetic tree based on internal transcribed spacer (ITS) sequences showing the phylogenetic placement for strain UR3 among next related fungal species. For detailed description see Supplementary Figure 1. The analysis was done accordingly. This analysis involved 71 nucleotide sequences and a total of 666 nucleotide positions in the final dataset. The sequence of *Rachicladosporium cboliae* was used as outgroup sequence to root the tree. Scale bars: nucleotide substitution per sequence position.

**Figure S3** Cytotoxic activity of the crude ethyl acetate extract of *Cladosporium* sp. UR3.


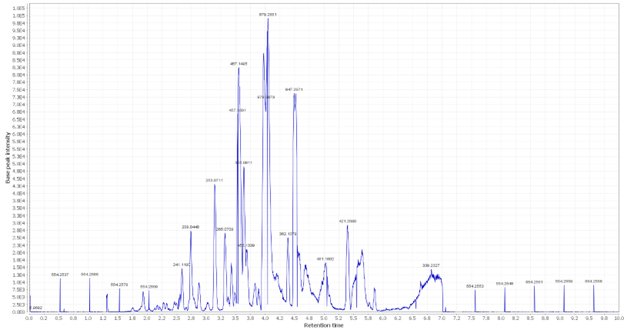
**Figure S4** The total ion chromatogram of the crude extract of *Cladosporium* sp. UR3 cultured on SDA media in negative ionization mode.


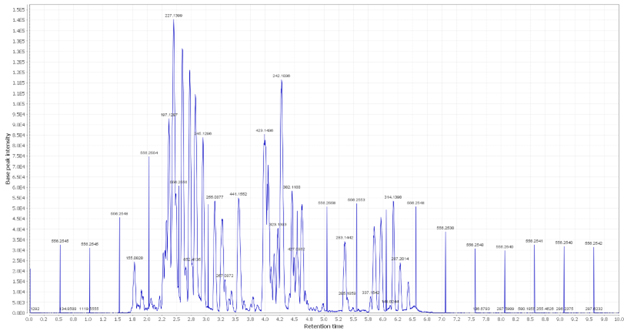


**Figure S5** The total ion chromatogram of the crude extract of *Cladosporium* sp. UR3 cultured on SDA media in positive ionization mode.

Table S1: Dereplicated metabolites from the ethyl acetate extract of *Cladosporium* sp. UR3.

| **no** | **m/z** | **M. Weight** | **Mass deference (ppm)** | **M. Formula** | **Name** | **Source** | **Ref.** |
| --- | --- | --- | --- | --- | --- | --- | --- |
| 1 | 141.0185 | 142.026 | 8 | C_6_H_6_O_4_ | Sumiki’s acid | *C. herbarum* | ^(^[^3^](#_ENREF_3)^)^ |
| 2 | 165.0545 | 166.062 | 8 | C_9_H_10_O_3_ | α-Acetylorcinol | *C. perangustm* | ^(^[^4^](#_ENREF_4)^)^ |
| 3 | 177.0549 | 178.062 | 8 | C_10_H_10_O_3_ | 4,8-Dihydroxy-1-tetralone |  |  |
| 4 | 179.071 | 180.078 | 7 | C_10_H_12_O_3_ | 1-(3,5-Dihydroxy-4-methylphenyl)propan-2-one |  |  |
| 5 | 195.0659 | 196.073 | 8 | C_10_H_12_O_4_ | Cladosporactone A | *C. cladosporioides* | ^(^[^5^](#_ENREF_5)^)^ |
| 6 | 209.0446 | 210.052 | 8 | C_10_H_10_O_5_ | Herbarin B | *C. herbarum* | ^(^[^6^](#_ENREF_6)^)^ |
| 7 | 229.1449 | 228.136 | 8 | C_12_H_20_O_4_ | iso-Cladospolide B | *C. herbarum* | ^(^[^3^](#_ENREF_3)^)^ |
| 8 | 233.0727 | 232.073 | 1 | C_13_H_12_O_4_ | Coniochaetone B | *C. halotolerans* | ^(^[^7^](#_ENREF_7)^)^ |
| 9 | 234.1128 | 235.120 | 8 | C_13_H_17_NO_3_ | Cladosporiumin I | *C. spherosperm-um* | ^(^[^8^](#_ENREF_8)^)^ |
| 10 | 235.0603 | 236.068 | 8 | C_12_H_12_O_5_ | Herbarin A | *C. herbarum* | ^(^[^6^](#_ENREF_6)^)^ |
| 11 | 245.1143 | 244.121 | 7 | C_14_H_16_N_2_O_2_ | (3*R*,8a*R*)-*Cyclo*(phenylalanylprolyl) | *C. cladosporioid-es* | ^(^[^9^](#_ENREF_9)^)^ |
| 12 | 249.1126 | 248.104 | 8 | C_14_H_16_O_4_ | Cladosporin C | *Cladosporium* sp. | ^(^[^10^](#_ENREF_10)^)^ |
| 13 | 261.040 | 262.047 | 7 | C_13_H_10_O_6_ | Coniochaetone K | *C. halotolerans* | ^(^[^7^](#_ENREF_7)^)^ |
| 14 | 269.0486 | 270.052 | 4 | C_15_H_10_O_5_ | Vertixanthone |  |  |
| 15 | 283.0715 | 284.068 | 3 | C_16_H_12_O_5_ | Methyl 8-hydroxy-6-methyl-9-oxo-9*H*-xanthene-1- carboxylate |  |  |
| 16 | 286.1526 | 285.157 | 5 | C_14_H_23_NO_5_ | Cladosporiumin H | *Cladosporium* sp. | ^(^[^11^](#_ENREF_11)^)^ |
| 17 | 291.1247 | 290.130 | 6 | C_20_H_18_O_2_ | Altertoxin IX | *Cladosporium* sp. | ^(^[^12^](#_ENREF_12)^)^ |
| 18 | 293.1297 | 292.131 | 2 | C_16_H_20_O_5_ | Malettinin B | *Cladosporium* sp*.* | ^(^[^13^](#_ENREF_13)^)^ |
| 19 | 299.0592 | 300.063 | 4 | C_16_H_12_O_6_ | Methyl 8-hydroxy-6-(hydroxymethyl)- 9-oxo-9*H*-xanthene-1-carboxylate | *C. halotolerans* | ^(^[^7^](#_ENREF_7)^)^ |
| 20 | 315.0539 | 316.058 | 5 | C_16_H_12_O_7_ | Conioxanthone A |  |  |
| 21 | 321.0882 | 320.089 | 1 | C_16_H_16_O_7_ | α-Diversonolic ester |  |  |
| 22 | 321.1241 | 322.120 | 4 | C_20_H_18_O_4_ | Altertoxin XII | *Cladosporium* sp*.* | ^(^[^12^](#_ENREF_12)^)^ |
| 23 | 337.0983 | 336.099 | 1 | C_20_H_16_O_5_ | Cladosporol H | *C. cladosporioid-es* | ^(^[^14^](#_ENREF_14)^)^ |
| 24 | 351.1286 | 352.131 | 3 | C_21_H_20_O_5_ | Cladosporol F |  |  |

**References**

1. Abdelmohsen UR, Cheng C, Viegelmann C, Zhang T, Grkovic T, Ahmed S, et al. Dereplication strategies for targeted isolation of new antitrypanosomal actinosporins A and B from a marine sponge associated-Actinokineospora sp. EG49. Marine drugs. 2014;12(3):1220-44.

2. <http://pubs.rsc.org/MarinLit/>. Septemper 2021.

3. Jadulco R, Proksch P, Wray V, Sudarsono, Berg A, Gräfe UJJonp. New Macrolides and Furan Carboxylic Acid Derivative from the Sponge-Derived Fungus Cladosporium h erbarum. 2001;64(4):527-30.

4. Fan Z, Sun Z-H, Liu H-X, Chen Y-C, Li H-H, Zhang W-MJJoAnpr. Perangustols A and B, a pair of new azaphilone epimers from a marine sediment-derived fungus Cladosporium perangustm FS62. 2016;18(11):1024-9.

5. He ZH, Zhang G, Yan QX, Zou ZB, Xiao HX, Xie CL, et al. Cladosporactone A, a unique polyketide with 7‐methylisochromen‐3‐one skeleton from the deep‐sea‐derived fungus Cladosporium cladosporioides. 2020;17(6):e2000158.

6. Jadulco R, Brauers G, Edrada RA, Ebel R, Wray V, Sudarsono, et al. New metabolites from sponge-derived fungi curvularia l unata and cladosporium h erbarum. 2002;65(5):730-3.

7. Wang C-N, Lu H-M, Gao C-H, Guo L, Zhan Z-Y, Wang J-J, et al. Cytotoxic benzopyranone and xanthone derivatives from a coral symbiotic fungus Cladosporium halotolerans GXIMD 02502. 2021;35(24):5596-603.

8. Liang X, Huang Z-H, Ma X, Qi S-HJMd. Unstable tetramic acid derivatives from the deep-sea-derived fungus Cladosporium sphaerospermum EIODSF 008. 2018;16(11):448.

9. Shaker N, Ahmed G, El-Sawy M, Ibrahim H, Ismail HJJoPP, Pathology. Isolation, characterization and insecticidal activity of methylene chloride extract of Cladosporium cladosporioides secondary metabolites against Aphis gossypii (Glov.). 2019;10(2):115-9.

10. Amin M, Zhang X-Y, Xu X-Y, Qi S-HJNpr. New citrinin derivatives from the deep-sea-derived fungus Cladosporium sp. SCSIO z015. 2020;34(9):1219-26.

11. Huang Z-h, Nong X-h, Liang X, Qi S-hJT. New tetramic acid derivatives from the deep-sea-derived fungus Cladosporium sp. SCSIO z0025. 2018;74(21):2620-6.

12. Zhang F, Zhou L, Kong F, Ma Q, Xie Q, Li J, et al. Altertoxins with quorum sensing inhibitory activities from the marine-derived fungus Cladosporium sp. KFD33. 2020;18(1):67.

13. Silber J, Ohlendorf B, Labes A, Wenzel-Storjohann A, Näther C, Imhoff JFJFiMS. Malettinin E, an antibacterial and antifungal tropolone produced by a marine Cladosporium strain. 2014;1:35.

14. Li H-L, Li X-M, Mándi A, Antus S, Li X, Zhang P, et al. Characterization of cladosporols from the marine algal-derived endophytic fungus Cladosporium cladosporioides EN-399 and configurational revision of the previously reported cladosporol derivatives. 2017;82(19):9946-54.
